# Respiratory Influenza A Virus Infection Triggers Local and Systemic Natural Killer Cell Activation *via* Toll-Like Receptor 7

**DOI:** 10.3389/fimmu.2018.00245

**Published:** 2018-02-13

**Authors:** Sabine Stegemann-Koniszewski, Sarah Behrens, Julia D. Boehme, Inga Hochnadel, Peggy Riese, Carlos A. Guzmán, Andrea Kröger, Jens Schreiber, Matthias Gunzer, Dunja Bruder

**Affiliations:** ^1^Immune Regulation, Helmholtz Centre for Infection Research, Braunschweig, Germany; ^2^Infection Immunology, Institute of Medical Microbiology, Infection Control and Prevention, Health Campus Immunology, Infectiology and Inflammation, Otto von-Guericke University, Magdeburg, Germany; ^3^Experimental Pneumology, University Hospital of Pneumology, University Hospital Magdeburg, Health Campus Immunology, Infectiology and Inflammation, Otto-von-Guericke University, Magdeburg, Germany; ^4^Department of Vaccinology and Applied Microbiology, Helmholtz Centre for Infection Research, Braunschweig, Germany; ^5^Molecular Microbiology, Institute of Medical Microbiology, Infection Control and Prevention, Health Campus Immunology, Infectiology and Inflammation, Otto-von-Guericke University, Magdeburg, Germany; ^6^Innate Immunity and Infection, Helmholtz Centre for Infection Research, Braunschweig, Germany; ^7^Institute for Experimental Immunology and Imaging, University Hospital, University of Duisburg-Essen, Essen, Germany

**Keywords:** influenza A virus, natural killer cells, pathogen-recognition receptors, Toll-like receptor 7, innate immunity, respiratory infection

## Abstract

The innate immune system senses influenza A virus (IAV) through different pathogen-recognition receptors including Toll-like receptor 7 (TLR7). Downstream of viral recognition natural killer (NK) cells are activated as part of the anti-IAV immune response. Despite the known decisive role of TLR7 for NK cell activation by therapeutic immunostimulatory RNAs, the contribution of TLR7 to the NK cell response following IAV infection has not been addressed. We have analyzed lung cytokine responses as well as the activation, interferon (IFN)-γ production, and cytotoxicity of lung and splenic NK cells following sublethal respiratory IAV infection in wild-type and TLR7ko mice. Early airway IFN-γ levels as well as the induction of lung NK cell CD69 expression and IFN-γ production in response to IAV infection were significantly attenuated in TLR7-deficient hosts. Strikingly, respiratory IAV infection also primed splenic NK cells for IFN-γ production, degranulation, and target cell lysis, all of which were fully dependent on TLR7. At the same time, lung type I IFN levels were significantly reduced in TLR7ko mice early following IAV infection, displaying a potential upstream mechanism of the attenuated NK cell activation observed. Taken together, our data clearly demonstrate a specific role for TLR7 signaling in local and systemic NK cell activation following respiratory IAV infection despite the presence of redundant innate IAV-recognition pathways.

## Introduction

Influenza A virus (IAV) is an Orthomyxovirus carrying a segmented, single-stranded RNA genome, and IAV infections remain a serious burden for human health during seasonal outbreaks. At the same time, there is a constant threat of newly emerging highly virulent pandemic strains. A full understanding of IAV pathogenesis and host responses will be crucial for optimizing the available prophylactic and therapeutic measures in the future.

Respiratory epithelial cells as well as alveolar macrophages, dendritic cells (DCs), and neutrophils are target cells of IAV (1, 2). Infected cells are able to recognize the virus through Toll-like receptor (TLR) 3, TLR7, RIG-I, MDA5, and the NLRP3 inflammasome, most of which sense the viral genome or replication intermediates (2) and contribute to the anti-IAV host defense in a cell-type-specific manner (3, 4). Several studies have addressed the role of TLR7 for host defense against IAV. Due to the presence of alternative innate IAV sensors and partially redundant signaling, TLR7ko mice respond to the virus and are able to survive the infection (5). Nevertheless, TLR7 has been found to affect several aspects of innate and especially adaptive B cell responses toward IAV (5–11).

Natural killer (NK) cells are innate lymphocytes that act as immune regulators through cytokine production and as cytotoxic effector cells. They are activated *in vivo* following IAV infection (12–14), and their main functions are the production of interferon (IFN)-γ and killing of infected host cells (15). However, the importance of NK cells in host defense against IAV is controversially discussed. Enhanced morbidity and mortality have been reported for mice depleted of NK cells and mice deficient of NKp46, an NK cell receptor that interacts with the IAV hemagglutinin (16, 17). By contrast, another study observed increased survival and ameliorated lung pathology in mice lacking NK cells (18). Ultimately, as recently shown, in mouse models, the contribution of NK cells to anti-IAV defense is strongly dependent on the viral strain and dose as well as the host-genetic background (14). Also for humans, the role of NK cells in IAV infection is not fully clarified, whereas recent studies from the 2009 H1N1 pandemic suggest a correlation between NK cell lymphopenia and disease severity (19–21). Interleukin-12 (Il-12), Il-15, Il-18, and type I IFN (IFN I) have been identified as upstream mediators of NK cell activation in viral infections (22–24). Following IAV infection, Il-12 contributes to early NK cell-dependent IFN-γ production in the respiratory tract (25), and IFN I has been shown to play a prominent role in IAV-mediated NK cell activation (12, 26, 27).

Interestingly, several studies have demonstrated potent TLR7-dependent NK cell activation by immunostimulatory RNAs in the context of antitumor immunity (28–35). However, the relevance of TLR7 signaling for the NK cell response mounted toward IAV infection has not been addressed so far. Therefore, we have studied this aspect of the anti-IAV immune response in TLR7-deficient hosts and indeed identified a distinct role for TLR7 in the IAV-mediated activation of NK cell effector function in the lung as well as in the periphery.

## Results

### The Lung NK Cell IFN-γ Response Mounted following IAV Infection Is Attenuated in TLR7ko Mice

**Figure 1:**
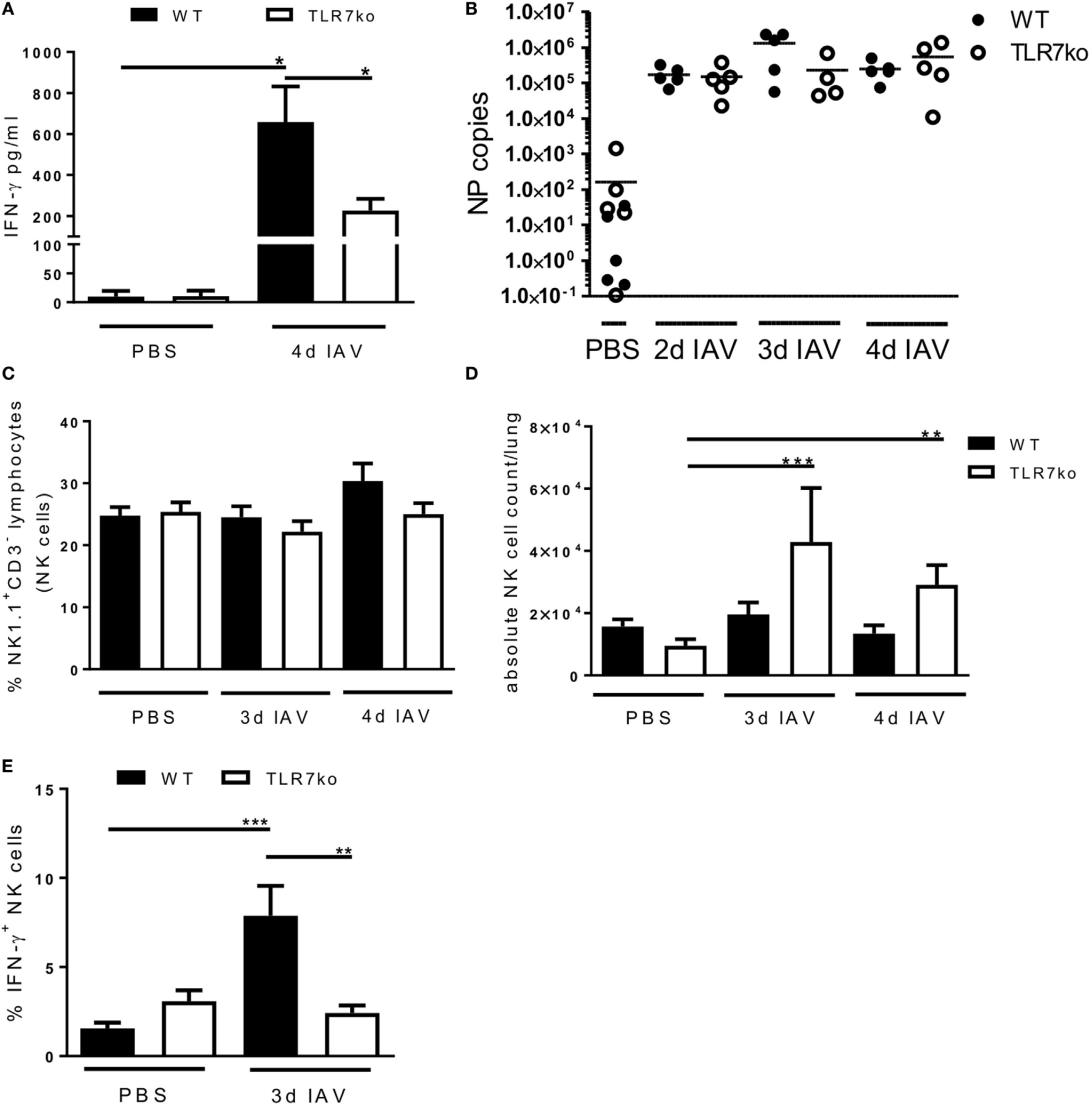
The early lung interferon-γ (IFN-γ) response following influenza A virus (IAV) infection is attenuated in toll-like receptor (TLR)7-deficient mice while the lung natural killer (NK) cell frequency is unchanged. Wild-type (WT) (black bars and symbols) and TLR7ko (open bars and symbols) mice were infected with 0.04 LD_50_ IAV PR8 or treated with PBS and sacrificed at the indicated time points. **(A)** IFN-γ levels in bronchoalveolar lavage were assessed by enzyme-linked immunosorbent assay. Data show the mean ± SEM of *n* = 3 uninfected and *n* ≥ 8 infected mice per strain with samples derived from three independent experiments. **(B)** IAV nucleoprotein (NP) copies were quantified in whole lung tissue cDNA as a measure for the viral load. Data show individual mice and the mean/group of samples collected from two independent infection experiments. **(C)** Lymphocytes isolated from the lung tissue were analyzed for the frequency of NK cells (CD3^−^/NK1.1^+^). Frequency ± SEM of NK cells within the lymphocyte population from *n* ≥ 6 mice per group compiled from at least two independent experiments. **(D)** Data show the absolute number of NK cells isolated per mouse lung for individual mice and the group mean compiled from at least two independent experiments. **(E)** Lymphocytes isolated from the lung tissue were analyzed for NK cell (CD3^−^/NK1.1^+^) IFN-γ production by intracellular flow cytometry *ex vivo*. Data show the frequency ± SEM of IFN-γ-positive NK cells within the NK cell population on day 3 post IAV infection for *n* ≥ 5 mice per group compiled from two independent experiments. Groups were compared by two-way ANOVA with Bonferroni multiple comparisons test (*p < 0.05, **p < 0.005, ***p < 0.0005).

In a previous study, we have characterized the respiratory anti-IAV response of TLR7ko mice and detected clearly reduced IFN-γ levels on day 3 and significantly reduced IFN-γ levels on day 5 post infection in comparison to that of wild-type (WT) hosts (11). As exclusively this early and not the later (day 7) IFN-γ response was affected and NK cells are typical early-acting producers of this cytokine, an underlying defect in NK cell activation was a likely cause. To further address this, we intranasally infected both WT and TLR7ko mice with a sublethal dose of IAV and confirmed reduced airway IFN-γ levels in TLR7ko mice on day 4 post infection (Figure 1A). Of note, the attenuated IFN-γ response was not a consequence of changes in the viral load between WT and TLR7ko mice (Figure 1B). Addressing the possible role of NK cells, we found that their frequency in the lung was not significantly altered between uninfected and infected or between WT and TLR7ko mice (Figure 1C). Nevertheless, a trend for a relative increase in the NK cell population in response to the infection was detectable in WT but not in TLR7ko mice on day 4 post infection (Figure 1C). Of note, the absolute number of lymphocytes isolated from the lungs on days 3 and 4 post IAV infection was not significantly altered between WT and TLR7ko mice (Figure S1A in Supplementary Material). Interestingly, however, on day 3 post infection, a significant increase in the absolute lymphocyte number, and on days 3 and 4 post infection, a significant increase in the absolute NK cell number were detectable in infected TLR7ko but not in WT mice (Figure S1A in Supplementary Material; Figure 1D). Therefore, the reduced local IFN-γ response detected in IAV-infected TLR7ko mice was not a consequence of a reduced number of NK cells present in the lungs. Nevertheless, we furthermore analyzed the production of IFN-γ by lung NK cells in response to IAV infection. Importantly, on day 3 post infection, there was a clear and significant increase in the frequency of IFN-γ producing NK cells in the lungs of WT but not in TLR7-deficient mice compared to uninfected controls (Figure 1E). Taken together, these data show a clear defect in lung NK cell IFN-γ production in TLR7-deficient mice early after IAV infection that was not a consequence of attenuated NK cell recruitment. This defect in NK cell function most likely underlays the reduced IFN-γ levels detected in TLR7ko mice early after IAV infection.

### The IAV-Mediated Induction of Lung NK Cell CD69 Expression Is Significantly Delayed in TLR7ko Mice Whereas Changes in the Maturation Status Are Not Affected

**Figure 2:**
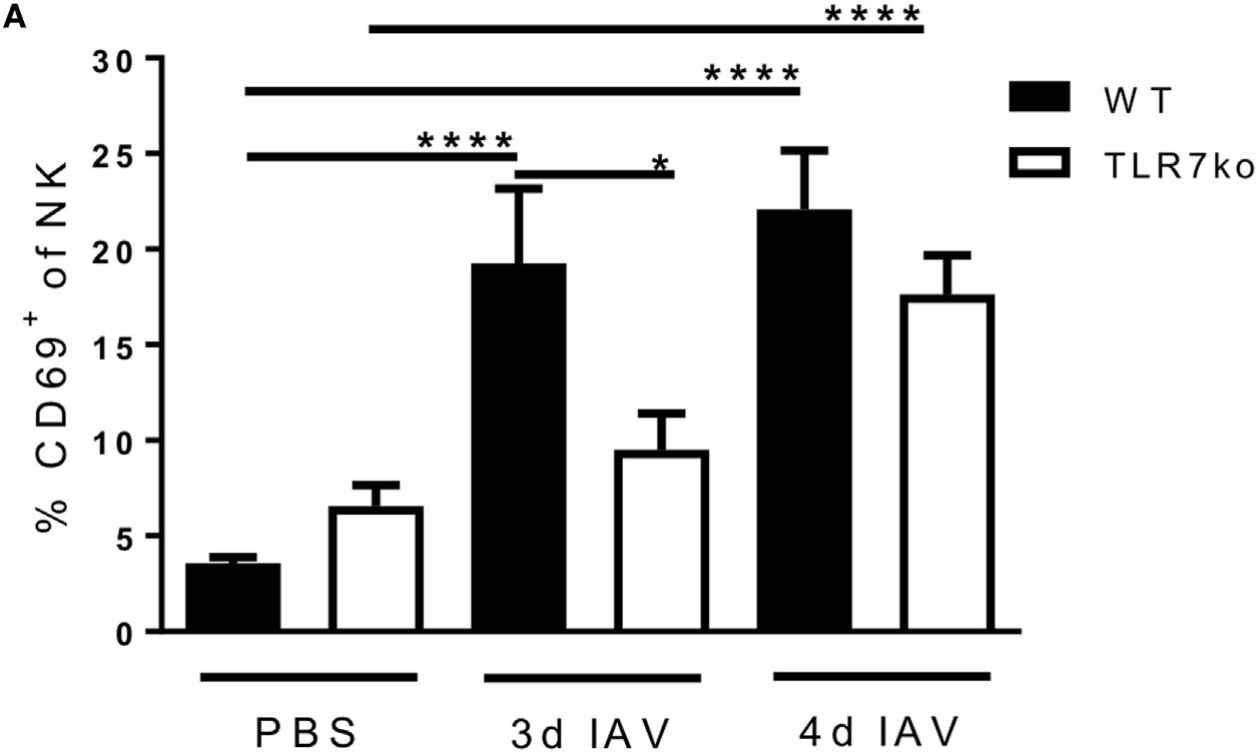

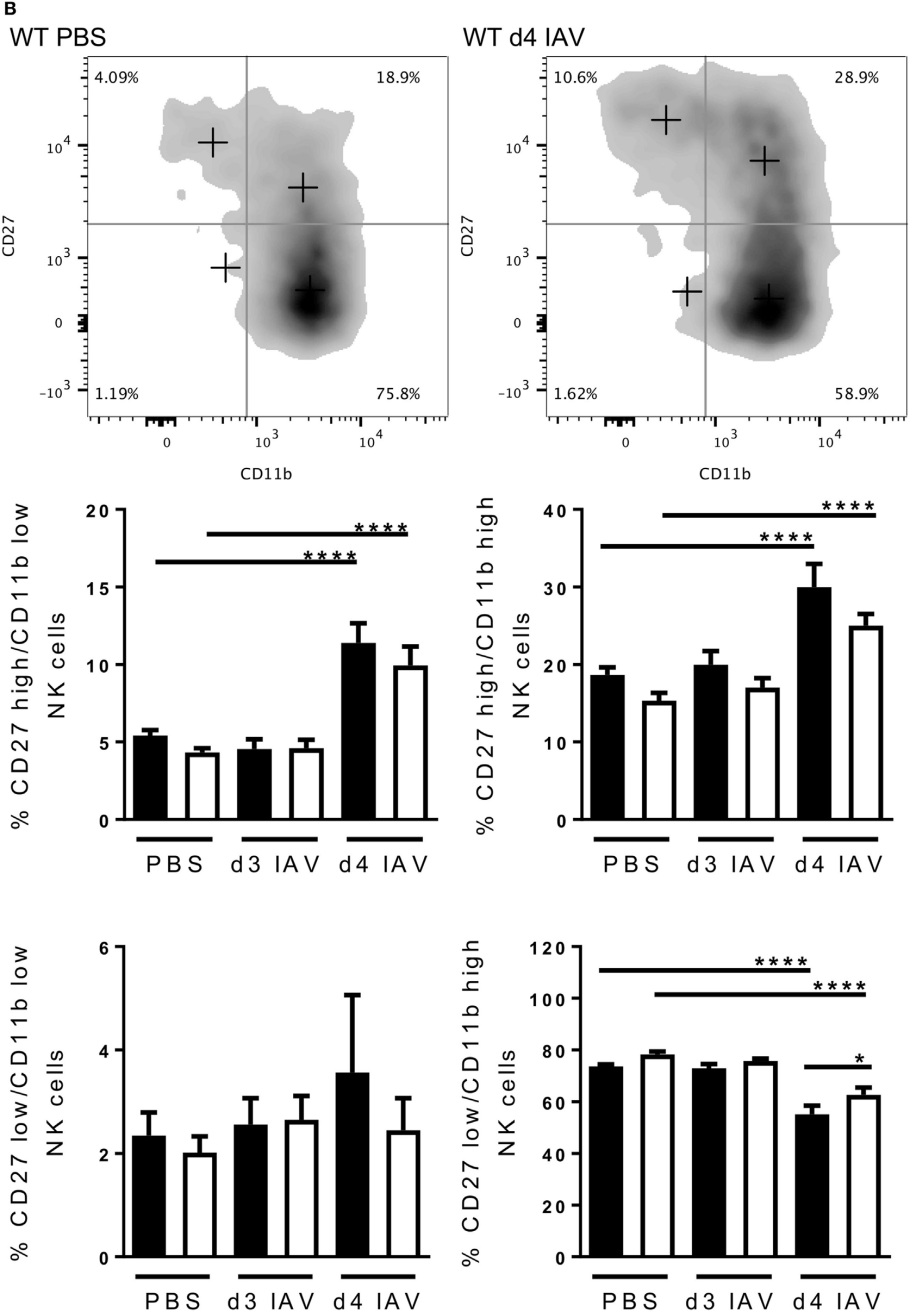
The induction of natural killer (NK) cell CD69 expression in the lung following respiratory influenza A virus (IAV) infection is delayed in Toll-like receptor (TLR)7-deficient hosts. Wild-type (WT) (black bars) and TLR7ko (white bars) mice were infected with 0.04 LD_50_ IAV PR8 or treated with PBS and sacrificed at the indicated time points. Lung NK cells (CD3^−^/NK1.1^+^) were analyzed for the expression of CD69 **(A)** and the co-expression of CD11b and CD27 **(B)** by flow cytometry. Data show the mean ± SEM of ≥6 mice/group from at last two independent experiments and representative flow-cytometric results for an uninfected and an IAV-infected WT mouse on day 4 post infection. Data were compared by two-way ANOVA with Bonferroni multiple comparisons test (*p < 0.05, ****p < 0.0001).

Based on these TLR7-dependent changes observed in the lung in the early phase of IAV infection, we further assessed the kinetics of NK cell activation at the site of infection by analyzing lung NK cells from IAV-infected WT and TLR7ko mice for the expression of the early activation marker CD69 (Figure 2A). Respiratory IAV infection potently induced CD69 expression by lung NK cells in WT mice on days 3 and 4 post infection. This was clearly delayed in TLR7ko mice, which displayed a significantly reduced frequency of activated CD69-expressing NK cells in the lung on day 3 post infection compared to WT mice. The induction of lung NK cell CD69 expression was, however, not fully TLR7-dependent, as significant CD69 expression was also detected in TLR7ko NK cells by day 4 post infection. In order to assess the activation status in more detail, we analyzed the co-expression of CD27 and CD11b on lung NK cells isolated from uninfected and IAV-infected WT and TLR7ko mice on days 3 and 4 post infection (36, 37) (Figure 2B). Compared to uninfected controls, respiratory IAV infection led to significant changes in the CD27/CD11b expression pattern in WT mice. While the frequency of the more mature CD27^high^/CD11b^low^ and CD27^high^/CD11b^high^ NK cell populations was significantly increased on day 4 post infection, the highly differentiated CD27^low^/CD11b^high^ NK cell population, which holds a higher activation threshold, was significantly decreased at this time point. As especially the co-expression of high levels of CD27 and CD11b is associated with potent effector functions (37), these data indicated IAV infection to trigger maturation and activation of NK cells in the lung. However, the CD27/CD11b expression pattern and its IAV-induced changes were largely unchanged between WT and TLR7ko mice following IAV infection. There was only a minor but significant increase in the highly differentiated CD27^low^/CD11b^high^ NK cell population on day 4 post infection in TLR7ko compared to that in WT mice. We also assessed the expression of NKp46, which can act as a receptor for the IAV hemagglutinin (16), on lung NK cells following IAV infection. However, NKp46 expression was only marginally changed following IAV infection without any significant changes between WT and TLR7ko mice (Figure S2A in Supplementary Material). Overall, our results regarding CD69, CD27, and CD11b expression on lung NK cells show a clear phenotypical activation and maturation in response to IAV infection that increased from days 3 to 4 post infection. Importantly, as NK cell IFN-γ production, also the timely induction of CD69 expression significantly depended on TLR7.

### Respiratory IAV Infection Activates Peripheral NK Cells in a TLR7-Dependent Manner

**Figure 3:**
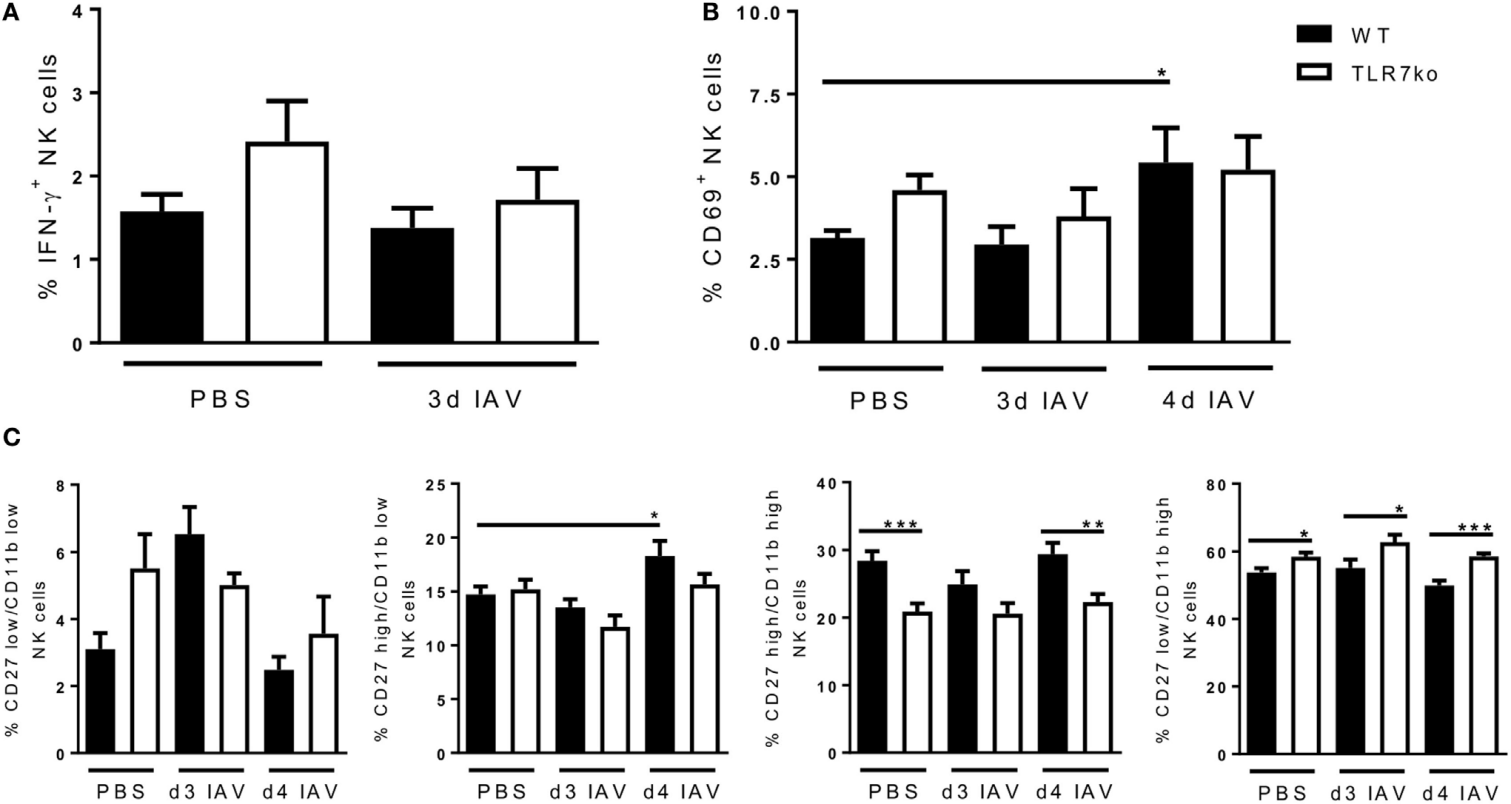
The expression of CD69 and CD27/CD11b by splenic natural killer (NK) cells in response to respiratory influenza A virus (IAV) infection is significantly altered in toll-like receptor (TLR)7-deficient mice. Wild-type (black bars) and TLR7ko (white bars) mice were infected with 0.04 LD_50_ IAV PR8 or treated with PBS and sacrificed at the indicated time points. Splenic NK cells were analyzed for the expression of intracellular interferon-γ (IFN-γ) **(A)** and surface CD69 **(B)** as well as CD11b/CD27 **(C)** by flow cytometry *ex vivo*. **(A)** Data show the mean ± SEM of 6 mice/group from two independent experiments. **(B)** Data show the mean ± SEM of ≥6 mice/group from at least two independent experiments. **(C)** Data show the mean ± SEM of ≥6 mice/group from at least two independent experiments. Data were compared by two-way ANOVA with Bonferroni multiple comparisons test (*p < 0.05, **p < 0.005, ***p < 0.0005).

Next to local NK cell activation at the site of infection, we further assessed the effect of respiratory IAV infection on systemic NK cells. While no induction of IFN-γ expression was detectable in splenic NK cells (Figure 3A), respiratory IAV infection induced a significant increase in CD69 expression on splenic NK cells in WT mice by day 4 post infection (Figure 3B). There was also a tendency for increased CD69 expression on splenic NK cells from infected TLR7ko mice, which was, however, not significant compared to the respective control group. Of note, basal CD69 expression detected in splenic NK cells of uninfected mice was elevated in TLR7ko mice. However, these changes were not significant. Regarding changes of the maturation status of peripheral NK cells in response to IAV infection, the more mature CD27^high^/CD11b^low^ NK cell population was significantly increased by day 4 post infection only in WT mice (Figure 3C). In contrast to lung NK cells, in the spleen, the frequency of both the CD27^low^/CD11b^high^ and the CD27^high^/CD11b^high^ NK cell populations was not changed following IAV infection in either WT or TLR7ko mice. Of note, there were significant changes of the CD27^high^/CD11b^high^ and the CD27^low^/CD11b^high^ NK cell populations TLR7ko mice that were independent of the infection. As for lung NK cells, NKp46 expression on splenic NK cells was only marginally changed following IAV infection without any significant changes between WT and TLR7ko mice (Figure S2B in Supplementary Material).

**Figure 4:**
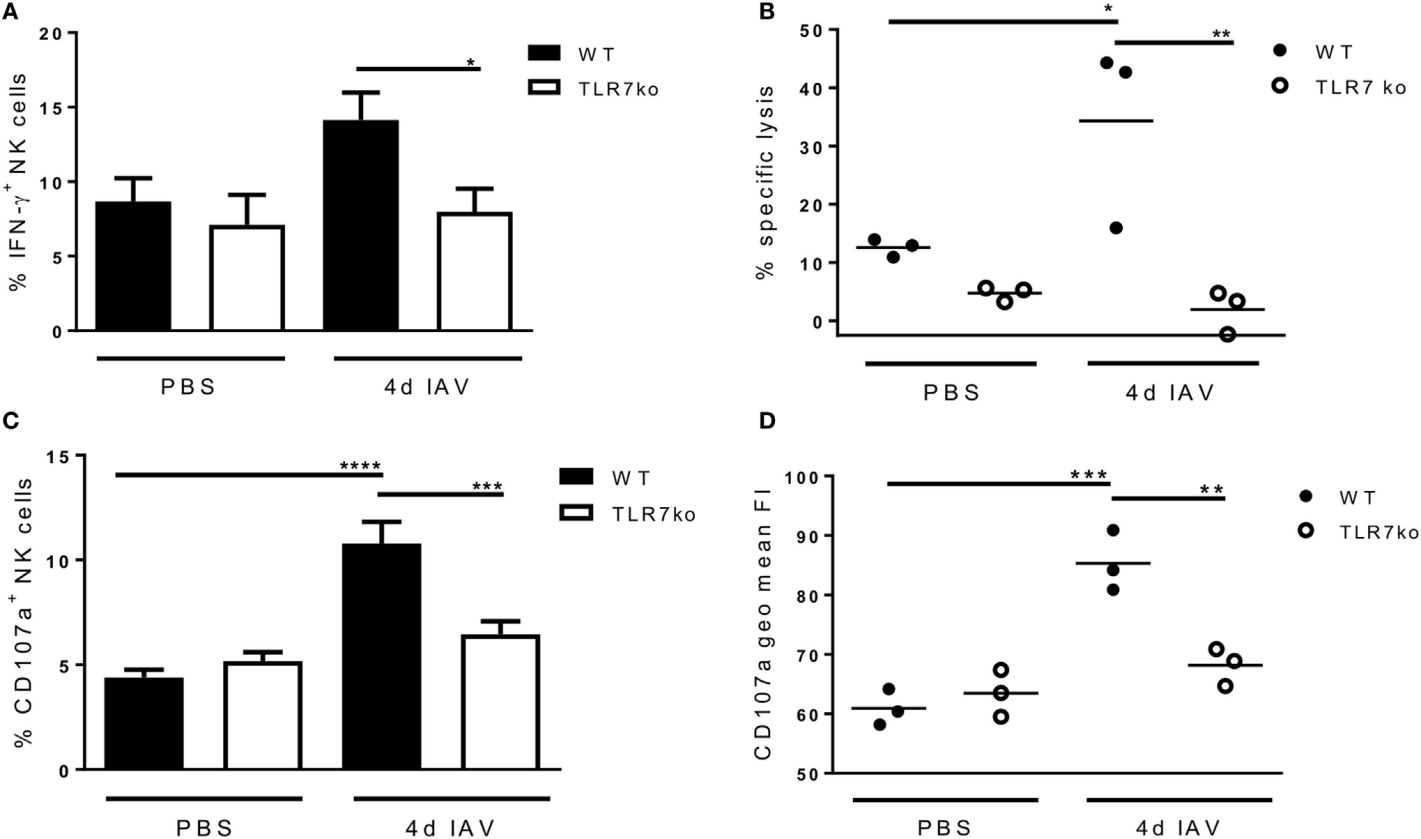
Respiratory influenza A virus (IAV) infection primes splenic natural killer (NK) cells for *in vitro* interferon-γ (IFN-γ) expression and target cell-directed cytotoxicity in a toll-like receptor (TLR)7-dependent fashion. Wild-type (WT) (black bars and symbols) and TLR7ko (white bars and symbols) mice were infected with 0.04 LD_50_ IAV PR8 or treated with PBS and were sacrificed on day 4 post infection. **(A)** The frequency of IFN-γ-positive NK cells (CD3^−^/NK1.1^+^) was quantified by flow cytometry following *in vitro* stimulation of splenocytes. Data represent the mean ± SEM of *n* = 6 mice from two independent experiments. **(B)** Specific target cell lysis in splenocyte/YAC-1 target cell co-cultures was assessed. Results show results for individual mice and the mean/group from one representative out of two independent experiments. **(C)** The frequency of CD107a^+^ NK cells (CD3^−^/NK1.1^+^) within splenocytes following co-incubation with YAC-1 target cells was assessed by flow cytometry. Data represent the mean ± SEM of *n* = 6 mice compiled from two independent experiments. **(D)** Geometric mean fluorescence intensity (FI) of the CD107a staining of the NK cell population assessed by flow cytometry following co-incubation of splenocytes with YAC-1 target cells. Results are shown for individual mice and the mean/group from one representative out of two independent experiments. Groups were compared by two-way ANOVA with Bonferroni multiple comparisons test (*p < 0.05, **p < 0.005, ***p < 0.0005, ****p < 0.0001).

**Figure 5:**
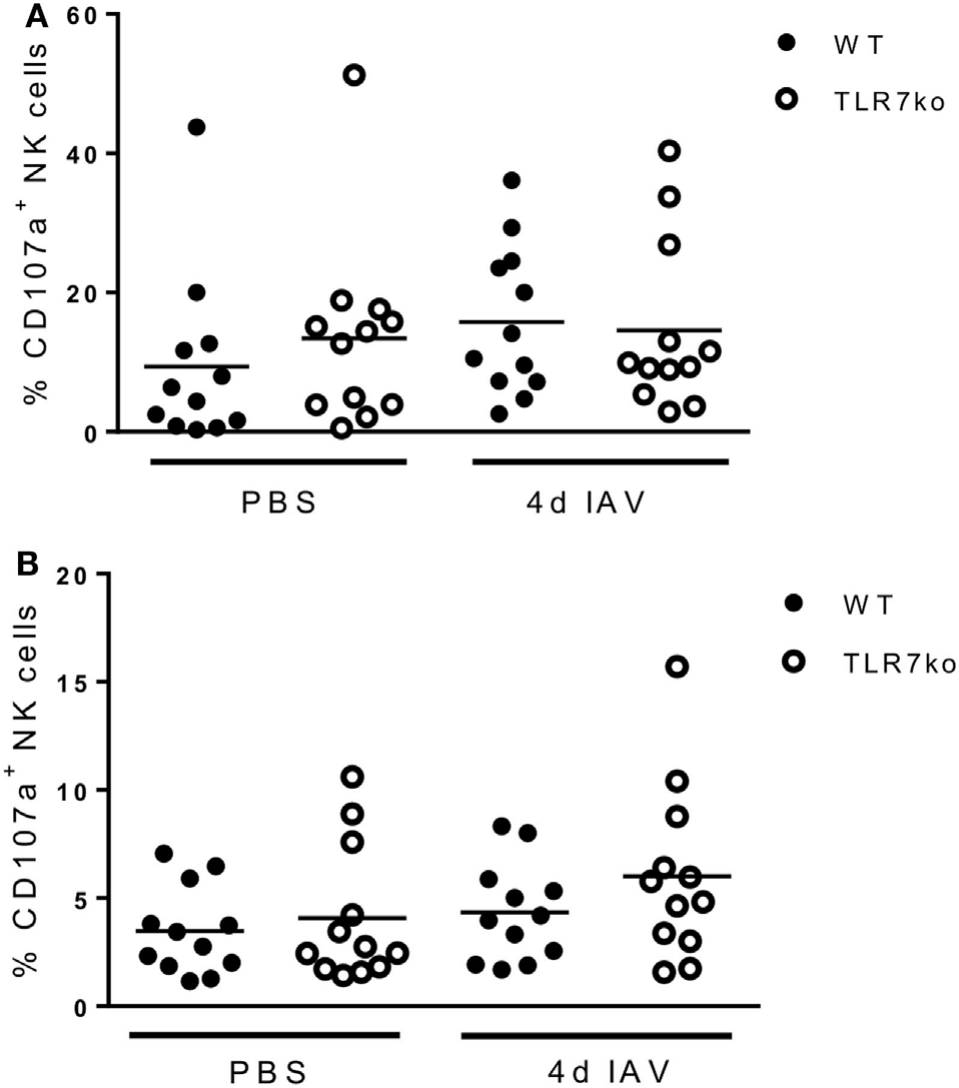
Respiratory influenza A virus (IAV) infection does not lead to significant degranulation of natural killer (NK) cells in the lung or spleen *in vivo*. Wild-type (WT) (black symbols) and toll-like receptor (TLR)7-deficient (open symbols) mice were infected with 0.04 LD_50_ IAV PR8 or treated with PBS and sacrificed on day 4 post infection. Lung **(A)** and splenic **(B)** NK cells were analyzed for surface CD107a expression by flow cytometry *ex vivo*. Data show the frequency of CD107a^+^ NK cells for 12 individual mice/groups compiled from four independent experiments. Data were compared by two-way ANOVA with Bonferroni multiple comparisons test.

Prompted by the significant activation of splenic NK cells that was detectable through increased *ex vivo* CD69 expression on day 4 following respiratory IAV infection, we additionally analyzed the effector functions of splenic NK cells isolated from IAV-infected hosts *in vitro* at that time point. Interestingly, following *in vitro* stimulation, more splenic NK cells isolated from IAV-infected WT mice expressed IFN-γ than those from uninfected mice. Importantly, significantly more IFN-γ producing splenic WT NK cells than TLR7ko splenic NK cells were detected (Figure 4A). Of note, there was no difference in the frequency of IFN-γ expressing lung NK cells isolated from IAV-infected WT and TLR7ko mice following *in vitro* stimulation, whereas the IFN-γ production on a per cell basis was significantly attenuated in TLR7ko NK cells (data not shown). As cytotoxicity displays a major effector function of NK cells and is induced in a TLR7-dependent manner by immunostimulatory RNA molecules (28), we assessed the potential of NK cells isolated from the spleens of IAV-infected WT and TLR7ko mice to specifically lyse target cells. Indeed, IAV infection triggered an increase in the specific lysis detected in splenocyte/target cell co-cultures, which was, however, only detectable in WT but not in TLR7-deficient hosts (Figure 4B). In addition, the accumulation of CD107a on the surface of NK cells in splenocyte/target cell co-cultures was analyzed as a measure for degranulation. In line with the detected target cell lysis, significant degranulation of splenic NK cells isolated from IAV-infected but not from control WT mice was detectable following co-culture with target cells. Both the proportion of CD107a^+^ NK cells (Figure 4C) and the amount of surface CD107a detected on NK cells (Figure 4D) were strongly and significantly increased in cells isolated from 4-day IAV-infected WT mice compared to those from uninfected controls. By stark contrast, no degranulation was detectable in splenic NK cells from IAV-infected TLR7ko mice following co-culture with target cells. Prompted by the strong induction of *in vitro* target cell-directed NK cell degranulation and cytotoxicity by respiratory IAV infection, we analyzed lung and splenic NK cells from IAV-infected WT and TLR7ko mice for degranulation *ex vivo*. However, we did not detect significant alterations to surface CD107a expression on NK cells isolated from the lung (Figure 5A) or the spleen (Figure 5B) 4 days post IAV infection compared to uninfected controls. Furthermore, there were no significant alterations in TLR7ko compared to WT mice regarding *ex vivo* NK cell CD107a expression. Taken together, these analyses clearly indicate that respiratory IAV infection triggers the systemic activation of NK cells. Importantly, in contrast to lung NK cells, IAV-induced CD69 expression, changes in the maturation status, *in vitro* IFN-γ production, and target cell-directed cytotoxicity of splenic NK cells were fully TLR7-dependent.

### The Il-12p40 and IFN I Response Is Attenuated in TLR7-Deficient Mice

**Figure 6:**
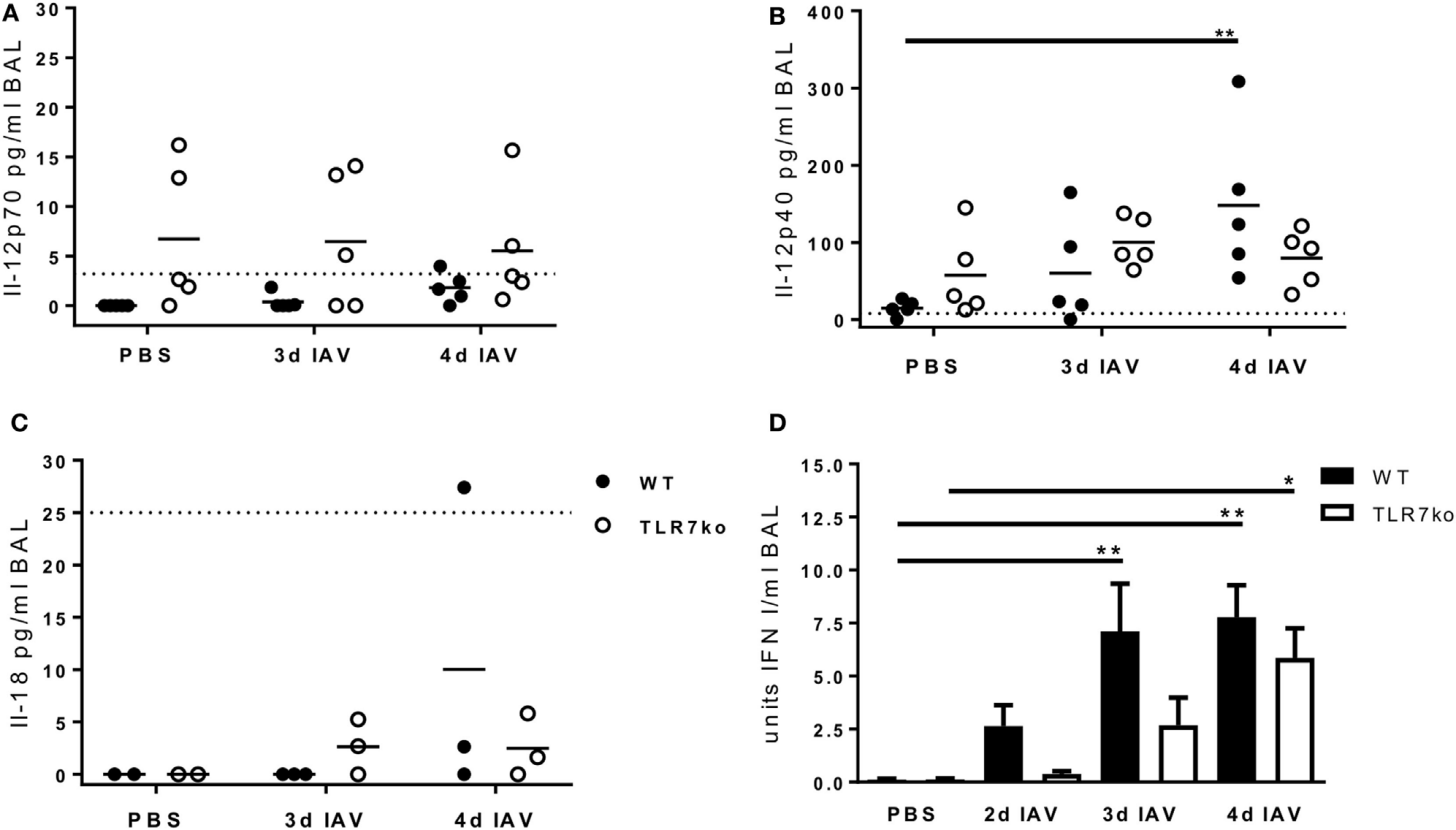
The early respiratory Il-12p40 and interferon (IFN) I response following influenza A virus (IAV) infection is attenuated in toll-like receptor (TLR)7-deficient mice. Wild-type (WT) (black bars and symbols) and TLR7ko mice (white bars and symbols) were infected with 0.04 LD_50_ IAV PR8 or treated with PBS and sacrificed at the indicated time points post infection. Il-12p70 **(A)**, Il-12p40 **(B)**, and Il-18 **(C)** were quantified in bronchoalveolar lavage (BAL). Data are shown for individual mice and indicate the mean/group of samples collected from at least two independent infection experiments. The dotted horizontal lines indicate the detection limit of the respective assay. **(D)** Bioactive IFN I was quantified in BAL. Data are shown as mean ± SEM of *n* ≥ 10 mice per group, and samples were collected in at least two independent infection experiments. Groups were compared by two-way ANOVA with Bonferroni multiple comparisons test (*p < 0.05, **p < 0.005).

Regarding the mechanisms of NK cell activation following IAV infection, Il-12 has been shown to contribute to early NK cell-dependent IFN-γ production (25), and especially IFN I has been identified as a key mediator of NK cell activation (12, 26, 27). Furthermore, also Il-15 and Il-18 have been implicated to play a role in IAV-mediated NK cell activation (12, 18, 38). In order to identify possible upstream defects that underlie the impaired NK cell activation in TLR7-deficient hosts following IAV infection, we assessed the levels of Il-12p70, Il-12p40, Il-18, and IFN I in the respiratory tract of IAV-infected WT and TLR7ko mice (Figure 6). Furthermore, we have previously characterized the respiratory cytokine response in WT and TLR7ko mice over the course of IAV infection in our model (11) and not detected Il-15 at any time point (data not shown). On days 3 and 4 post infection, there was no induction of Il-12p70 in comparison to uninfected mice (Figure 6A). By contrast, a significant production of Il-12p40 was detectable in WT mice by day 4 post infection, whereas only a marginal and not significant increase in Il-12p40 was detected in TLR7-deficient mice (Figure 6B). At the same time, no increase in Il-18 levels was detectable in the respiratory tract of WT or TLR7ko mice on days 3 and 4 post IAV infection (Figure 6C). As IFN I is produced very rapidly following viral infections and as we have previously described a significantly impaired very early respiratory IFN I response in TLR7ko mice following lethal IAV infection (39), we now assessed the very early kinetics of respiratory IFN I production following sublethal infection on days 2, 3, and 4 post infection (Figure 6D). Indeed, a significant delay in the early IFN I response of TLR7ko mice became apparent. An efficient and significant induction of IFN I was detectable from day 3 post infection in WT mice but only by day 4 in TLR7ko mice, and the mean IFN I levels were reduced in IAV-infected TLR7ko compared to those in WT mice at all tested time points. Of note, we also analyzed systemic Il-12, Il-18, and IFN I levels with regard to the systemic NK cell activation observed following IAV infection, but did not detect any increase in the serum levels in response to IAV infection at any of the time points analyzed. Of note, there was, however, a significant decrease in serum Il-12p40 in TLR7ko mice following infection (Figure S3 in Supplementary Material). Ultimately, the attenuated Il-12p40 and the impaired and delayed IFN I response observed in the airways of TLR7ko mice following sublethal IAV infection correlated well with their attenuated NK cell response.

## Discussion

Prompted by the attenuated early respiratory IFN-γ response of TLR7ko mice following sublethal IAV infection, our study addressed the role of TLR7 signaling in IAV-mediated NK cell activation. Indeed, we discovered a clear contribution for TLR7 in the induction of a timely local and systemic NK cell response following IAV infection.

In line with previous studies (12–14, 21, 26), we detected significant activation of NK cells following respiratory IAV infection through CD69 expression by lung and splenic NK cells and IFN-γ production by lung NK cells *ex vivo*. *In vitro*, we detected clear cytotoxicity and degranulation of splenic NK cells from IAV-infected mice in response to target cells as well as IFN-γ production in response to unspecific stimulation. Interestingly, no significant but only very marginal degranulation of NK cells was detectable *ex vivo* following isolation from the site of infection. While cytotoxicity and degranulation of lung NK cells from IAV-infected mice toward target cells has been shown *in vitro* (12, 26, 38), our findings are in line with a report by others showing no changes in *ex vivo* surface CD107a expression over the early course of IAV infection in C57Bl/6 mice (14). Ultimately, this lack of detectable degranulation at the site of infection suggests that cytokine production rather than cytotoxicity is the predominant effector function of lung NK cells following IAV infection. Regarding CD27/CD11b expression, we found the majority of lung and also splenic NK cells in uninfected mice to display a more functionally mature phenotype, as has also been reported by others (13, 40). NK cell differentiation has been described to subsequently progress from CD27^low^/CD11b^low^ over CD27^high^/CD11b^low^ and CD27^high^/CD11b^high^ stages to CD27^low^/CD11b^high^. Here, the CD11b^high^ populations are reported to possess more potent effector functions, and of these the CD27^high^/CD11b^high^ NK cell population displays the most potent effector cells (36). In line with a previous report (40), we observed a clear shift toward the less differentiated but more functionally mature phenotypes for both lung and splenic NK cells in the course of IAV infection, supporting the concept of site-specific maturation in the periphery (41). Importantly, we detected a strong increase in the CD27^high^/CD11b^high^ effector NK cell population in the lung by day 4 post IAV infection, reflecting IAV-induced activation of NK cells at the site of infection. Surprisingly and in contrast to other reports, however (14), we did not observe a significant increase in the overall population size or the absolute NK cell numbers in the lungs of WT mice during the first 4 days post IAV infection despite the rapid and strong activation of NK cells. Surprisingly and in contrast to WT mice, we found NK cell counts to be significantly increased in infected versus uninfected TLR7ko mice, possibly displaying a compensatory mechanism for attenuated NK cell activation. Ultimately, the details of recruitment of NK cells of different maturation states and/or the proliferation and local maturation at the site of IAV infection as well as the discrimination between tissue-resident and peripheral NK cells will have to be addressed specifically by future investigations.

Most importantly, our study shows that in a number of aspects, the activation of both local and systemic NK cells in response to respiratory IAV infection significantly depends on TLR7. The production of IFN-γ by lung NK cells and the induction of CD69 expression by lung and splenic NK cells triggered by respiratory IAV infection were clearly affected in TLR7-deficient hosts. Importantly, the priming for *in vitro* IFN-γ production and target-cell-directed cytotoxicity of splenic NK cells through respiratory IAV infection was fully dependent on TLR7. This finding is well in line with the TLR7-dependent induction of NK cell antitumor activity through immunostimulatory RNA molecules (28, 35, 42). The role of TLR7 in IAV-mediated NK cell activation has, however, not been addressed so far, and our study now shows that also IAV infection activates and primes for NK cell effector function in a TLR7-dependent manner. This specific role for TLR7 is remarkable in the light of the fact that viral infections trigger broader and more versatile immune responses compared to the treatment with immunostimulatory RNAs or even specific TLR ligands. Mechanistically, NK cell activation through immunostimulatory molecules has been described to mainly depend on the presence of DCs that signal to NK cells *via* Il-12 and IFN I (28, 33, 42, 43). Regarding the mechanisms of NK cell activation following IAV infection, contributing roles have been described for Il-15 (12, 18), Il-12 (25, 27), Il-18 (38), and IFN I (12, 26, 27, 44), with IFN I displaying the key player (12, 44). As for the induction of NK cell antitumor activity by immunostimulatory molecules, activation following IAV infection has been demonstrated to occur in an accessory cell-dependent manner (27, 44), with a major function for plasmacytoid DC (pDC) (44). Interestingly, the production of large amounts of IFN I by pDC is a major function specific for TLR7, also following IAV infection (10, 45, 46). Accordingly, we have previously described a delayed and attenuated IFN I response in the lungs of TLR7ko mice following lethal IAV infection (39) even though TLR7-deficient hosts are generally able to mount IFN I responses following viral infection *via* alternative receptors (5). Importantly, here we show a significantly delayed IFN I response in TLR7-deficient mice following sublethal IAV infection that correlates with the observed defects in NK cell activation. Most likely, there is a mechanistic link *via* TLR7-dependent IAV recognition and IFN I production by pDC. Nevertheless, TLR7ko mice did mount an IFN I response comparable to the WT by day 4 post infection, well in line with the delayed but nevertheless significant activation of lung NK cells also in these mice. Furthermore, we show an attenuated Il-12p40 response in the airways of TLR7ko mice post infection that possibly also contributes to the attenuated NK cell activation observed in the lung. Generally, NK cell activation, i.e., the significant induction of CD69 expression and also the shift toward a higher frequency of CD27^high^CD11b^low^ NK cells, was stronger at the site of infection than in the periphery. This observation possibly explains why in splenic NK cells, CD69 expression and the frequency of CD27^high^CD11b^low^ cells were significantly increased only in infected WT and not in TLR7ko mice and only by day 4 post infection. Importantly, we did, however, not detect any increase in Il-12, Il-18, or IFN I in serum samples of infected WT or TLR7ko mice, leaving open the questions of whether minimal but not detectable early systemic IFN I or Il-12 originating from the lung and present only in WT mice due to a threshold effect was sufficient to activate splenic NK cells, whether there are alternative fully TLR7-dependent mechanisms of systemic NK cell activation, or whether this issue is a matter of recirculation of NK cells locally activated in the lung to the periphery (47). Ultimately, also the question regarding the contribution of direct viral recognition by NK cells, which do express TLR7 (33, 48), to their activation following IAV infection arises. For a first insight into this question, we adoptively transferred TLR7-deficient NK cells into WT recipients and analyzed their CD69 and IFN-γ expression in the lung following IAV infection (Figure S4 in Supplementary Material). There was little activation of TLR7ko NK cells in the lungs of WT recipients following IAV infection, which was, however, not as clear and strong as in WT NK cells adoptively transferred to WT recipients as control. These data suggest that both accessory cell-dependent and direct activation *via* TLR7 play a role in TLR7-dependent IAV-mediated NK cell activation, and the detailed contributions will need to be clarified in future investigations. Though mostly not significant, to our knowledge, we are the first to report altered baseline CD69 as well as CD27/CD11b expression of lung and splenic NK cells in uninfected TLR7ko compared to that in uninfected WT mice, and therefore also a contribution of intrinsic effects of TLR7-deficiency on NK cell maturation and activation to the observations made cannot fully be excluded. Nevertheless, our data show a clear role of TLR7 in the timely and efficient activation of NK cells in response to IAV infection. A number of studies of the past, including our own, have demonstrated TLR7 to play only a minor role in viral clearance and survival to IAV infection but to rather fine-tune innate responses (5, 8, 11, 39). Also, the overall significance of NK cells for anti-IAV responses has not been conclusively clarified [reviewed in Ref. (15)]. In line with this, our results suggest NK cells not to be crucial for anti-IAV defense in a sublethal challenge, as the defects in NK cell activation observed in TLR7-deficient mice did not show effects on the viral load. However, next to ensuring viral clearance, NK cells have been described to serve important functions in shaping adaptive antiviral responses in general (23, 49, 50) and also specifically in IAV infections (51, 52). Importantly, TLR7 has also been shown to be significantly involved in the development of fully functional adaptive immune reactions, also in the context of vaccination and persistent viral infection (5, 6, 9, 53–55). Therefore, delayed and incomplete NK cell activation possibly contributes to the defects in adaptive immune responses observed in TLR7-deficient mice following IAV infection. Severe secondary bacterial infections are a major complication of IAV infections, and we have previously shown a benefit for TLR7ko mice in antibacterial clearance following IAV/*Streptococcus pneumoniae* coinfection (11). As IFN-γ is known to have detrimental effects in coinfections (56, 57), the attenuated NK cell IFN-γ response observed in TLR7ko mice correlates well to this finding, and the modulation of antibacterial defense in secondary bacterial infection displays a possible downstream effect of NK cell activation following IAV infection.

Taken together, our study adds a novel piece of knowledge to our understanding of the induction of NK cell responses following IAV infection. Importantly, we have identified a previously unrecognized specific function of TLR7. Such knowledge will ultimately be essential for a full understanding of IAV pathogenesis and in turn for the development of efficient prophylactic and therapeutic measures.

## Materials and Methods

### Mice

All experiments were performed in female mice 8−12 weeks of age with an average weight of 23 g. TLR7-deficient mice (58) (provided by S. Bauer) were bred at the animal facility of the Helmholtz Centre for Infection Research (HZI) and were backcrossed to the C57BL/6J background for a total of 10 generations. C57BL/6J mice were obtained from Harlan (now Envigo) or bred at the HZI.

### IAV Infection

Madin–Darby canine kidney cell-derived IAV PR8/A/34(H1N1) was obtained as described previously (59). Following anesthesia through inhalation of isoflurane or intraperitoneal injection of ketamine–xylazine, mice were intranasally infected with 0.04 MLD_50_ diluted in PBS. Control animals were treated with PBS.

### Isolation of Lymphocytes from the Respiratory Tract and Spleen

Lungs were once flushed through the trachea with 1 ml cold sterile PBS to obtain bronchoalveolar lavage (BAL). Lavaged lungs were perfused with PBS, excised and minced on ice, followed by enzymatic digestion for 45 min at 37°C in Iscove’s modified Dulbecco’s medium containing 0.2 mg/ml Collagenase D (Roche), 0.01 mg/ml DNase (Sigma-Aldrich), and 5% fetal calf serum. After the addition of EDTA (5 mM final concentration), suspensions were filtered (70 µm) and pelleted by centrifugation. Enrichment for lymphocytes was performed using Ficoll–Paque PLUS (GE Healthcare Life Sciences) or Easycoll (1.124 g/ml; Merck Millipore) following erythrocyte lysis by osmotic shock. Splenocytes were isolated by homogenization of spleens through a 70-µm cell strainer in PBS using a syringe plunger, centrifugation of the cell suspension, and erythrocyte lysis by osmotic shock.

### Stimulation for Intracellular IFN-γ Staining

Lymphocytes isolated from the lung tissue were incubated with 10 ng/ml Phorbol 12-Myristate 13-Acetate (PMA) (Sigma-Aldrich) and 1 µg/ml Ionomycin (Sigma-Aldrich) for 4 h; 5 µg/ml Brefeldin A (Sigma-Aldrich) was added after the first 2 h of incubation. Splenocytes were incubated in a medium containing 10 ng/ml PMA for 5 h, and 5 µg/ml Brefeldin A was added after 1 h.

### YAC-1 Target Cell Co-Incubation

Isolated splenocytes were counted and co-incubated with YAC-1 target cells at a 10:1 ratio at 37°C and 5% CO_2_. After 1 h, 0.006 µg/ml Monensin and 5 µg/ml Brefeldin A (Sigma-Aldrich) were added. After a total of 5 h, cells were stained for flow cytometry.

### Flow Cytometry

Cell suspensions were incubated with a CD16/CD36 (2.4G2) antibody for the blocking of Fc receptors and stained with fixable live/dead stain (Molecular Probes) as indicated. Antibody stainings on the cell surface were performed for CD3 (145-2C11), NK1.1 (PK136), NKp46 (29A1.4), CD11b (M1/70), CD27 (LG.7F9), CD69 (H1.2F3), and CD107a (1D4B). Intracellular staining for IFN-γ (XMG1.2) was performed following fixation with 2% paraformaldehyde and permeabilization with 0.1% Igepal CA-630 (Sigma-Aldrich). Data were acquired using a BD Fortessa and analyzed using FlowJo (Tree Star). Following exclusion of dead cells and gating on singlets and lymphocytes, NK cells were defined as the CD3^−^/NK1.1^+^ population. For the calculation of absolute cell numbers, 20,000 polystyrene beads (Comp Beads Plus negative control beads, BD Biosciences) were added to each sample, and absolute cell numbers were calculated in relation to the acquired bead population.

### ^51^Cr Release Assay for the Detection of Specific Target Cell Lysis

Splenocytes isolated at day 4 post infection were used as effector cells at a concentration of 1 × 10^6^/ml and co-cultured with ^51^chromium-labeled YAC-1 cells, a well-known target for NK cells. YAC-1 target cells were incubated in RPMI (Invitrogen Life Technologies) without FCS and labeled with 100 μCi of ^51^Cr (Amersham) for 1.5 h at 37°C and 5% CO_2_. Effector and target cells were incubated at an effector/target ratio of 10:1 for 4 h at 37°C and 5% CO_2_. Subsequently, cells were centrifuged, and the radioactivity present in the supernatant was measured by scintillation counting. Spontaneous lysis was detected in untreated target cells, whereas the maximal lysis was determined after adding 100 µl of 5% Triton X-100 (Carl Roth). Results are expressed as the percentage of lysed cells according to the calculation {(sample − spontaneous lysis)/[(maximal lysis − spontaneous lysis) × 100]}.

### Quantification of Cytokines

Bronchoalveolar lavage samples were centrifuged for 10 min at 4°C and 10,000 × *g*. Il-18, IFN-γ, Il-12/Il-23(p40), and Il-12(p70) were detected by enzyme-linked immunosorbent assay (ELISA) according to the manufacturer’s recommendations (MBL for Il-18, BioLegend ELISA Max for all others). For the quantification of IFN I, IFN-sensitive epithelial cells from Mx2-Luc reporter mice were treated with BAL or serum and analyzed for luciferase activity as previously described (38, 60).

### Quantification of the Viral Load

The viral load in lung tissue homogenates was determined as IAV nucleoprotein (NP) RNA copies by absolute qRT-PCR. Perfused lung tissue was stored in RNAlater solution (Ambion), and RNA was extracted using the RNA-easy kit (Qiagen) following homogenization using a manual disperser (Kinematica). For cDNA synthesis 1 μg of RNA was transcribed using the Maxima First Strand cDNA Synthesis Kit for RT-qPCR (Thermo Scientific). Absolute qRT-PCR was performed on a LightCycler 480 II (Roche) using FastStart Essential DNA Green Master (Roche). Per reaction 25 ng reversely transcribed RNA was used and compared to a plasmid standard containing defined copy numbers of the IAV NP gene. NP primers were GAGGGGTGAGAATGGACGAAAAAC (5'-NP) and CAGGCAGGCAGGCAGGACTT (3'-NP) and were used in a final concentration of 500 nmol/l.

### Statistical Analyses

Groups were compared by the indicated statistical test using the GraphPad Prism software, and *p* ≤ 0.05 was considered indicative of statistical significance.

## Ethics Statement

This study was carried out in accordance with the recommendations of national and international guidelines. The protocol was approved by the “Niedersächsisches Landesamt für Verbraucherschutz und Lebensmittelsicherheit.”

## Author Contributions

SS-K designed and performed experiments and wrote the manuscript; SB, JB, and IH performed experiments; PR designed and performed experiments; AK designed experiments and provided research materials; CG and JS provided research materials; MG designed experiments and provided research materials; DB designed experiments, provided research materials, and wrote the manuscript.

## Conflict of Interest Statement

The authors declare that the research was conducted in the absence of any commercial or financial relationships that could be construed as a potential conflict of interest.
