# Supplementary material for: Respiratory Influenza A Virus Infection Triggers Local and Systemic Natural Killer Cell Activation via Toll-Like Receptor 7
Source: Front Immunol. 2018 Feb 13;9:245. doi: 10.3389/fimmu.2018.00245 (PMC5819576; doi:10.3389/fimmu.2018.00245)
Supplement: Supplementary file 1 [file image_1.PDF]

A

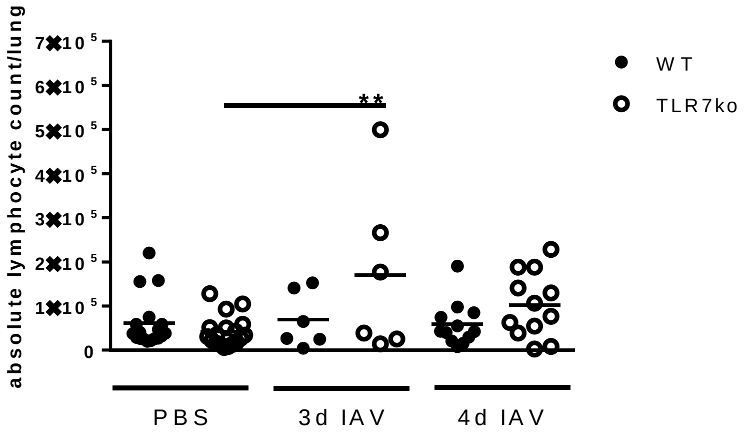

Supplementary figure 1. Absolute lymphocyte counts detected in the lungs of WT and TLR7ko mice on days 3 and 4 post IAV infection. WT and TLR7ko mice were infected with 0.04 LD_{50} IAV PR8 or treated with PBS and sacrificed at the indicated time-points. Lung cells were isolated as described in materials and methods. For the quantification of absolute cells numbers, a defined number of polystyrene particles (7.5 µm) (Becton Dickinson, CompBeads Plus) were added to each sample before staining for flow cytometry. Absolute cell numbers in the samples were calculated from the ratio of the number of beads added over the bead count acquired and the cell count acquired. Data show the absolute number of lymphocytes **(A)** isolated per mouse lung for individual mice and the group mean compiled from at least 2 independent experiments. Groups were compared by two-way ANOVA with Bonferroni multiple comparisons test (** $p < 0.01$).

A

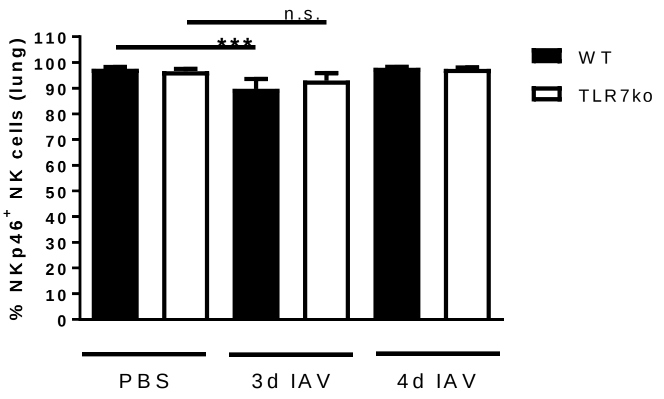

B

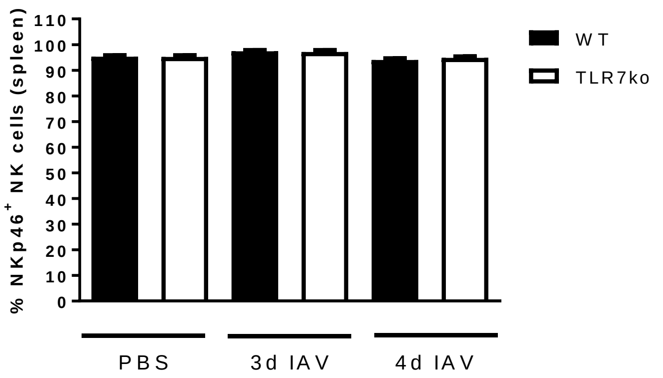

Supplementary figure 2. NKp46 expression of lung and splenic NK cells following respiratory IAV infection. WT and TLR7ko mice were infected with 0.04 LD_{50} IAV PR8 or treated with PBS and sacrificed at the indicated time-points. The expression of NKp46 on NK cells ($CD3^-$/$NK1.1^+$) from lungs **(A)** and spleens **(B)** was analyzed by flow cytometry. Data show the mean ± SEM of n ≥ 6 mice per group compiled from at least two independent experiments. Groups were compared by two-way ANOVA with Bonferroni multiple comparisons test (n.s. = not significant, *** $p < 0.0005$).

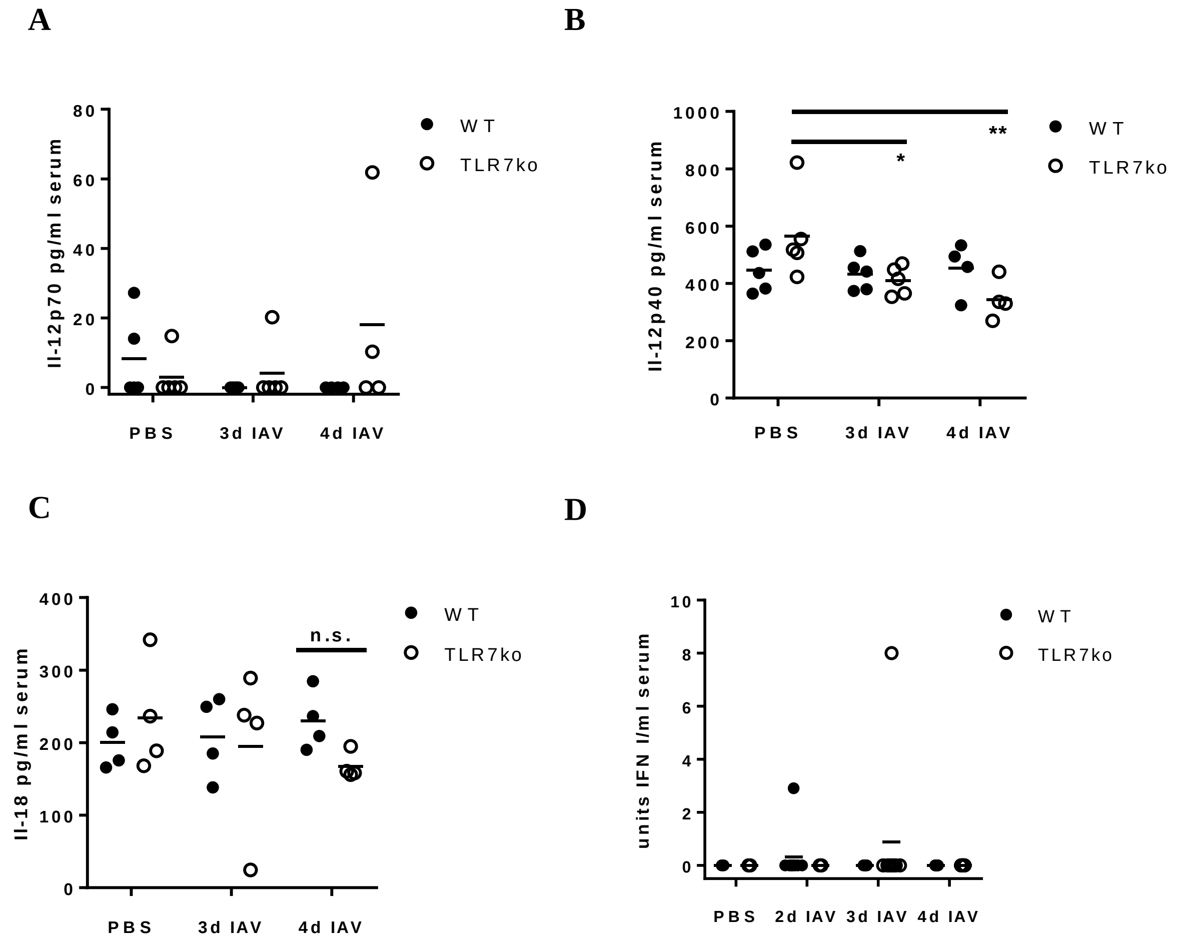

Supplementary figure 3. Serum levels of Il-12p70, Il-12p40, Il-18 and IFN I. WT (filled circles) and TLR7ko (open circles) mice were infected with 0.04 LD50 IAV PR8 or treated with PBS and sacrificed at the indicated time-points post infection. Il-12p70 **(A)**, Il-12p40 **(B)** and Il-18 **(C)** were quantified in serum by ELISA. Data are shown for individual mice with the mean/group of samples collected in at least two independent infection experiments. **(D)** Bioactive IFN I was quantified in serum. Data are shown for individual samples with the mean/group of n = 5 uninfected and n = 9 infected mice per group. Samples were collected in at least two independent infection experiments. Groups were compared by two-way ANOVA with Bonferroni multiple comparisons test (n.s. = not significant, * $p < 0.05$, ** $p < 0.005$).

A

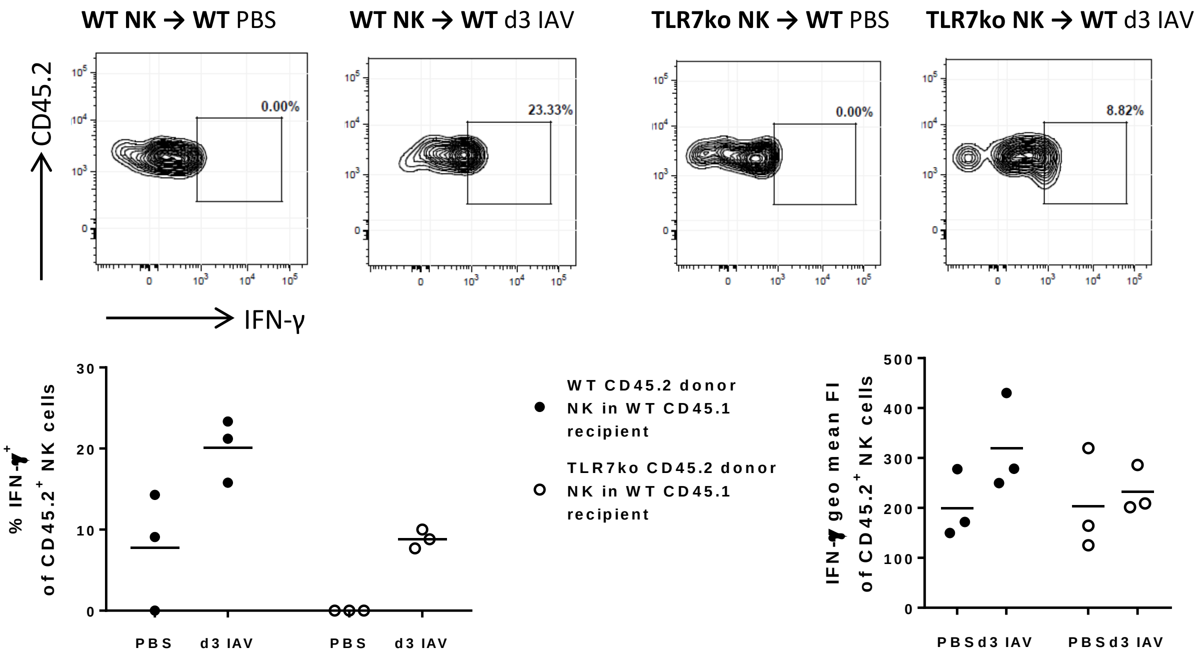

B

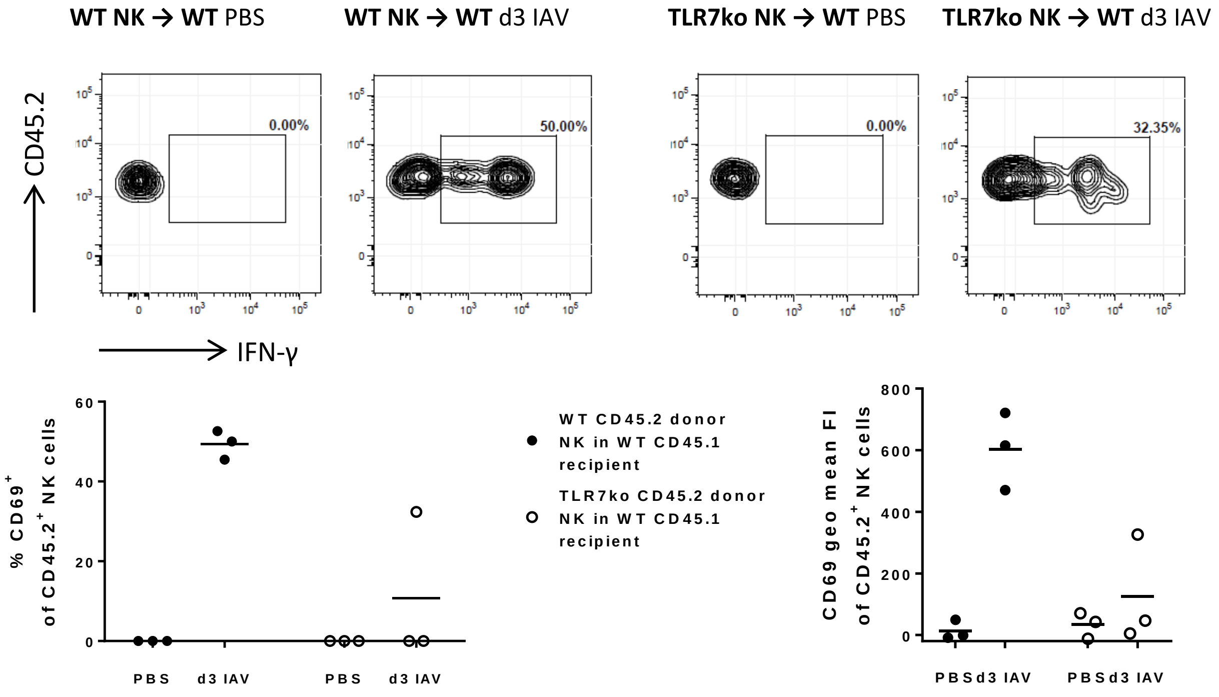

C

Sample	$CD45.2^+$ NK cell count
1 WT NK → WT PBS	18
2 WT NK → WT PBS	11
3 WT NK → WT PBS	7
4 TLR7ko NK → WT PBS	13
5 TLR7ko NK → WT PBS	4
6 TLR7ko NK → WT PBS	19

Sample	$CD45.2^+$ NK cell count
7 WT NK → WT d3 IAV	30
8 WT NK → WT d3 IAV	57
9 WT NK → WT d3 IAV	33
10 TLR7ko NK → WT d3 IAV	10
11 TLR7ko NK → WT d3 IAV	34
12 TLR7ko NK → WT d3 IAV	13

Supplementary figure 4. Transfer of NK cells isolated from uninfected WT or TLR7ko mice into congenic, IAV infected WT recipients. Untouched NK cells were enriched from 5 x 10^8 splenocytes of uninfected WT and TLR7ko mice using the MACS Miltenyi NK cell isolation kit according to the manufacturer's recommendations. NK cell frequency was assessed and was 55 - 60% following enrichment. $CD45.1^+$ congenic recipient C57Bl/6J mice were infected with 0.04 LD_{50} IAV PR8 or treated with PBS and immediately i.v. injected with 2.6 x 10^5 WT NK cells or 9.4 x 10^5 TLR7ko NK cells, respectively. On day 3 post infection, mice were sacrificed and lung lymphocytes were analyzed by flow cytometry. Cells were stained with a fixable live/dead stain (Molecular Probes) and stained with antibodies specific for CD45.2, CD3 and NK1.1 to identify the transferred donor NK cells ($CD45.2^+$, $NK1.1^+$, $CD3^-$). Donor NK cells were analyzed for intracellular IFN-γ **(A)** and surface CD69 expression **(B)**. FACS plots show $CD3^-/NK1.1^+$ NK cells from one representative mouse per group. Graphs show summarized data for the frequency of IFN-γ^+ (A) or $CD69^+$ (B) donor NK cells as well as the geometric mean of the fluorescence intensity for the IFN-γ as well as CD69 staining for all detected donor NK cells. Data are shown for individual mice and the mean/group for one experiment with three mice per group. **(C)** The tables show the number of donor NK cells ($CD45.2^+$ $CD3^-NK1.1^+$) that could be detected and were analyzed.
